# Allelopathic Activity of Ginseng-Cultivated Soil: Extracts on Seed Germination and Growth of Five Vegetables in China

**DOI:** 10.3390/plants15111607

**Published:** 2026-05-23

**Authors:** Jun Lei, Tianyi Wang, Wei Lin, Zhengwu Liu, Jiaqi Yang, Wanting Niu, Zichu Zhao, Jiarui Chen, Ping Chen, Yi Wang

**Affiliations:** 1 College of Life Science, Jilin Agricultural University, Changchun 130118, China; leijun3000@163.com (J.L.);; 2 Jilin Engineering Research Center Ginseng Genetic Resources Development and Utilization, Changchun 130118, China

**Keywords:** ginseng-cultivated soil, seed germination, seedling growth, antioxidant enzyme, photosynthetic pigments

## Abstract

Allelopathy means that one plant produces chemical substances to affect the growth of other plants. Crop rotation is considered as a potential strategy to alleviate the allelopathic inhibition. So, it is important to identify rotation crops with wide availability and low inhibitory effects. In this study, the allelopathic potential of soil extracts was investigated on the germination, seedling growth, biomass, and biochemical parameters (malondialdehyde, photosynthetic pigments, and antioxidant enzyme activities) of five crops, by a series of laboratory experiments. Firstly, both soil water extracts (SWE) and soil ethanol extracts (SEE) exhibited allelopathic inhibition on the seed germination and the root length of all seedlings in a dose-dependent relationship. The SWE significantly promoted the shoot length of bok choy and Chinese lettuce, while the SEE had no significant effect in bok choy. The application of SEE resulted in a significant increase in the dry weight of bok choy and rocket. In contrast, SWE had a negligible effect on bok choy and lettuce. Both of them caused decrease in the dry weight of the other seedlings. Then, the allelopathic synthetic effect index of water/ethanol extracts was chemo-inhibitory, and the inhibitory effect increased with increasing extract concentration. The SWE had the strongest inhibition on rocket and the SEE on lettuce. Both of them had the weakest effect on bok choy. The extracts significantly inhibited the photosynthetic capacity in five crops, manifested as decrease in photosynthetic pigments and dose-dependent effects. The malondialdehyde (MDA) content in all crops increased in a dose-dependent manner, confirming that the extracts caused lipid peroxidation. However, the defense strategies of different crops vary significantly. There is crop with active defense, such as bok choy treated with SWE. It delayed oxidative damage by continuously upregulating the activities of superoxide dismutase (SOD) and catalase (CAT). This is the key physiological mechanism for tolerance. There is also the oxidative stress failure type, as follows: CAT activity of rocket and cabbage increased, but the SOD activity did not increase by SEE. This reveals the physiological essence of their sensitivity—the lack of persistent scavenging ability for reactive oxygen species. Based on the inhibition of peroxidase (POD) and ascorbic acid peroxidase (APX), it is speculated that the extracts may inhibit the hydrogen peroxide scavenging pathway, which centered on the ascorbate–glutathione cycle. It is the fundamental reason why the continuous accumulation of MDA though SOD/CAT is up. This study confirmed the allelopathic effects of the water and ethanol extracts on five vegetable crops, and found that bok choy was less affected by them. The soil extracts affected the growth and development of seedlings by regulating their oxidative metabolism and photosynthetic capacity. These results support recommending pak choi as a rotation crop. This provides crops for subsequent field experiments and a new direction for next-step research on continuous cropping obstacles.

## 1. Introduction

Ginseng, known as a traditional Chinese medicine, is the dried root and rhizome of the plant *Panax ginseng* C.A. Meyer, which is a member of the Araliaceae family. It has the effects of enhancing immunity [1], combatting fatigue [2,3], improving cardiovascular function [4], possessing anti-tumor properties [5], and delaying aging [6].

Ginseng is mainly distributed in Jilin, Liaoning, and the eastern of Heilongjiang Province in China, as well as parts of North Korea and Russia. It is one of the genuine medicinal materials in Jilin Province. Cultivated ginseng is commonly referred to as “garden ginseng”. Wild forest ginseng is called “mountain-cultivated ginseng” [7]. In the early days, forests were cut down and ginseng was planted in forest soil in China. This led to a shortage of suitable land and severe ecological damage. In recent years, China has prohibited planting in newly deforested forest soil. As a result, the ginseng cultivation pattern has changed. Farmland has become the main method in major ginseng-producing areas in China.

However, continuous cultivation of ginseng on the same plot can change soil properties. These include physical and chemical properties, enzyme activity, and microbial communities [8,9,10]. This practice often results in increased pathogenic bacteria [11], exacerbated diseases, reduced emergence rates, and decreased yields [12]. This phenomenon is called continuous cropping obstacle, which seriously affects the development of ginseng industry. The causes of this obstacle are complex, involving the deterioration of soil physical and chemical properties, accumulation of auto toxic substances, and imbalance in soil microecology. There are traditional methods for dealing with continuous cropping obstacles. These methods include soil improvement [13,14], application of bio-bacterial fertilizers, and soil disinfection [15,16]. However, these methods lead to farmland occupation and soil pollution. Therefore, it is necessary to find a new method to solve the problem of continuous ginseng cultivation.

Numerous studies indicate that allelochemicals may be responsible for plant toxicity and soil microecological degradation [17,18]. Additionally, allelopathic autotoxicity is a significant factor contributing to the continuous cropping issue in ginseng cultivation [19]. Research indicates that phenolic acids are one of the main allelochemicals in ginseng. Some phenolic acids have been verified for auto-toxic effects such as *p*-coumaric acid, salicylic acid, cinnamic acid, vanillic acid, syringic acid, benzoic acid, vanillin, ferulic acid, *p*-Hydroxybenzoic acid, gallic acid, and 3-phenylpropanoic acid [20,21,22,23]. Triterpenoid saponins are also one of the main allelopathic auto toxic substances in ginseng, including ginsenosides Rb_1_, Rb_2_, Rd, Rg_1_, Rg_2_, Re, and R_1_ [12,24,25,26]. Once these substances accumulate in the soil, they not only directly inhibit the seed germination and root growth of ginseng, but also induce oxidative stress responses, which in turn disrupt the structure of cell membranes. This disruption leads to an increase in malondialdehyde (MDA) levels, which further impacts the normal physiological metabolism of plants [20].

Crop rotation can change the quality of litter and the composition of root exudates by increasing plant diversity, thus alleviating problems such as microecological imbalance. Some studies have shown that crop rotation can increase soil nutrient content, alter soil microbial community diversity [27], and reduce the incidence of soil-borne diseases, thereby improving crop yields and restoring soil health [28]. Compared with continuous monoculture, crop rotation can effectively alleviate continuous cropping obstacles.

Pot experiments showed that the fresh weight of ginseng roots increased and root activity was enhanced after rotating with *Perilla frutescens* (L.) Britt. [29]. The rotation of ginseng and celandine (*Chelidonium majus* L.) significantly increased soil pH, nutrient content, and enzyme activity, which indicated an improvement in soil fertility [30]. Planting Astragalus membranaceus and Perilla frutescens in ginseng soil improved the nutrient imbalance and soil microecology [31]. Microbial diversity was improved with the increase in rotation crops. The cultivation of Medicago sativa and Perilla frutescens significantly changed the composition of soil microbiota and reduced the abundance of potential pathogenic bacteria [32]. Therefore, screening suitable rotation crops is an important issue to be solved at present.

This study systematically evaluated the effects of water and ethanol extracts on the seed germination and seedling growth of bok choy, rocket, cabbage, lettuce, and Chinese lettuce by a series of laboratory experiments. The allelopathic effects were determined by biological indicators such as root length, shoot length, and dry and wet weights, and the allelopathic mechanism was judged by physiological and biochemical indicators. Crops with non-significant allelopathic inhibition were screened as alternative plants for crop rotation with ginseng.

## 2. Results

### 2.1. Allelopathic Effects of Water/Ethanol Extracts from Soil

To explore the allelopathic effects of two extracts of soil, seed germination and seedling growth of bok choy, rocket, cabbage, lettuce, and Chinese lettuce were investigated after treatment of extracts.

#### 2.1.1. Effects of Soil Extracts on Seed Germination

**Figure 1:**
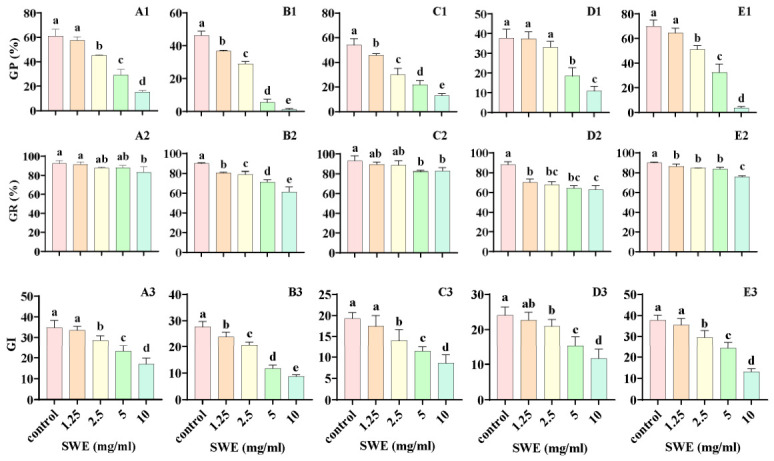
Effects of SWE on Seed Germination of Five Vegetable. (**A**) bok choy, (**B**) rocket, (**C**) cabbage, (**D**) lettuce, (**E**) Chinese lettuce, (**1**) GP, (**2**) GR, (**3**) GI. The comparison between multiple samples was determined by One-way ANOVA using Tukey’ s test, and the different letters (a–e) indicate significant differences (*p* < 0.05).

**Figure 2:**
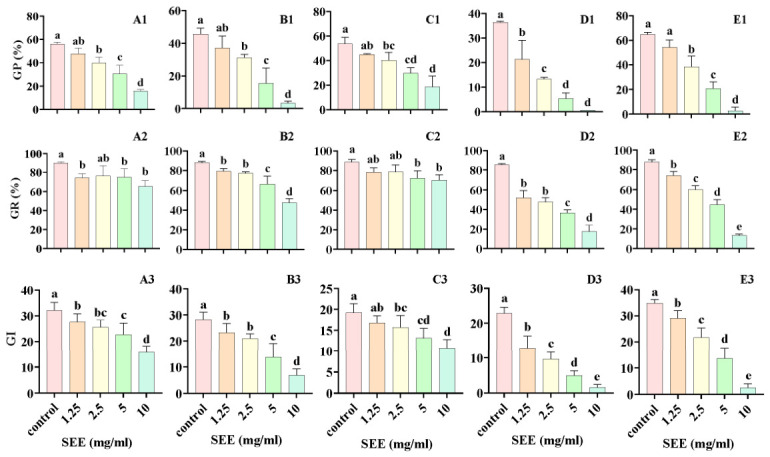
Effects of SEE on Seed Germination of Five Vegetables. (**A**) bok choy, (**B**) rocket, (**C**) cabbage, (**D**) lettuce, (**E**) Chinese lettuce, (**1**) GP, (**2**) GR, (**3**) GI. The comparison between multiple samples was determined by One-way ANOVA using Tukey’ s test, and the different letters (a–e) indicate significant differences (*p* < 0.05).

When seeds were cultured with extracts in a range of concentrations (2.5~10 mg/mL), soil water extracts (SWE) and soil ethanol extracts (SEE) exerted different effects on the seed germination of five plants (Figure 1 and Figure 2 and Table 1 and Table 2). Rocket was most sensitive to SWE, with significant inhibition in germination potential (GP), germination rate (GR), and germination index (GI). Bok choy showed the highest tolerance, with significant inhibition at 10 mg/mL (GR) and 2.5 mg/mL (GP and GI). Cabbage was the least affected by SEE, showing significant differences from the control at 2.5 mg/mL for GP and GI, at 5 mg/mL for GR, whereas GR and GI in the other seeds were significantly inhibited at 1.25 mg/mL. Allelopathy inhibition of SEE was stronger than that of SWE for the same crop.

#### 2.1.2. Effects of Soil Extracts on Seedling Growth

**Figure 3:**
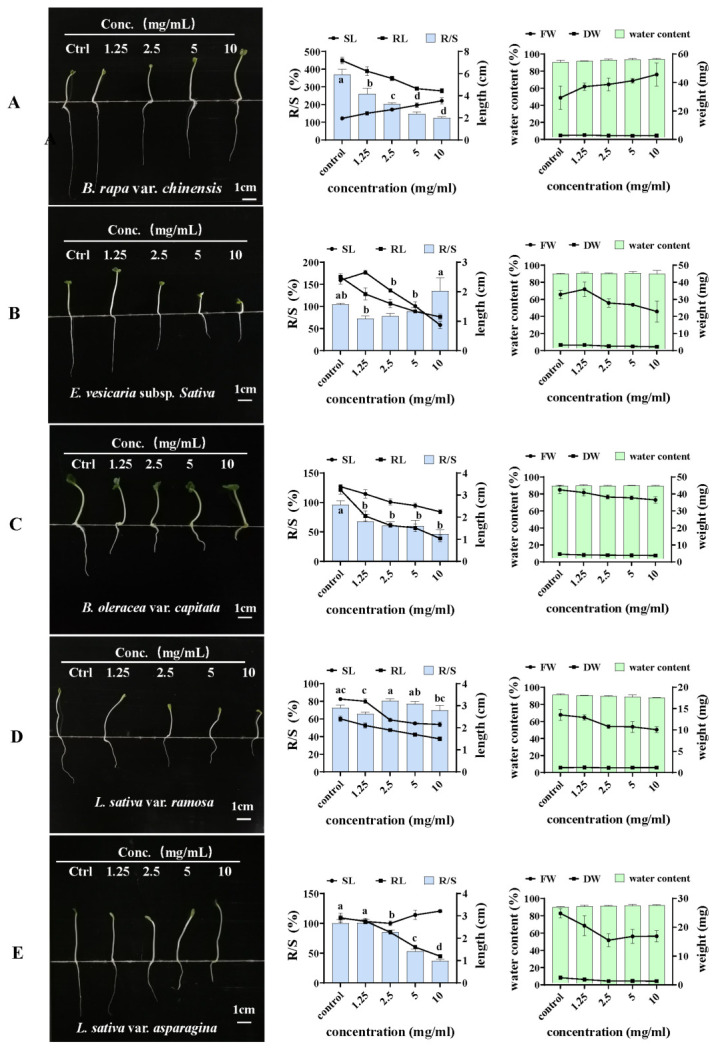
Effects of SWE on seedling growth. (**A**) bok choy, (**B**) rocket, (**C**) cabbage, (**D**) lettuce, (**E**) Chinese lettuce. The comparison between multiple samples was determined by One-way ANOVA using Tukey’ s test, and the different letters (a–d) indicate significant differences (*p* < 0.05). All the water content had no significant differences.

**Figure 4:**
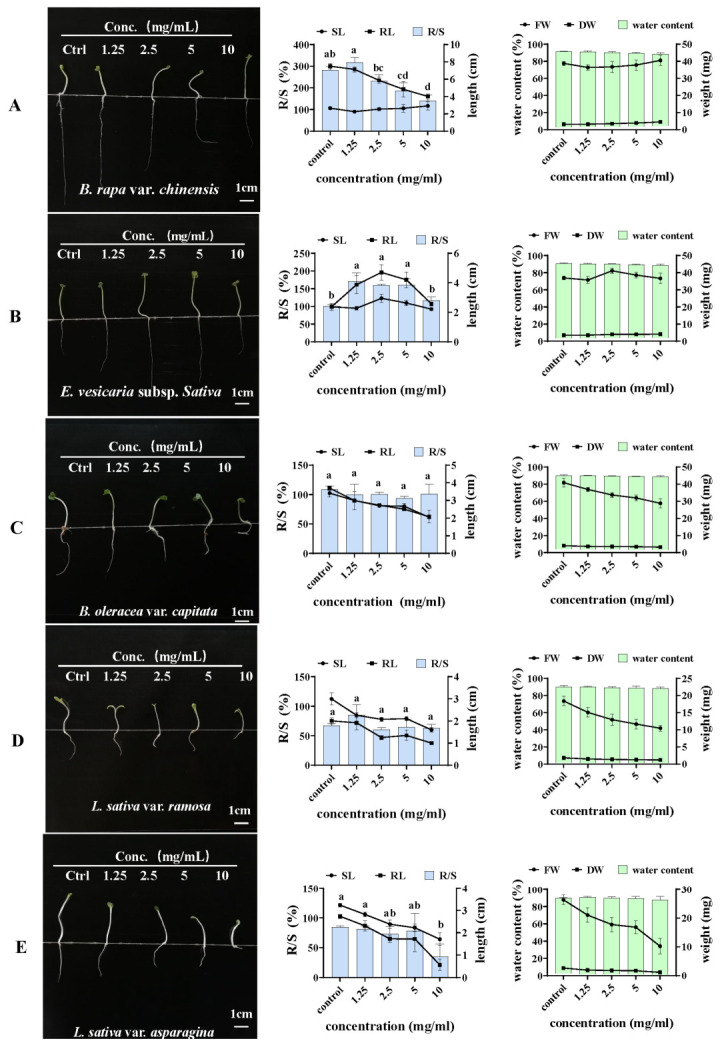
Effects of SEE on seedling growth. (**A**) bok choy, (**B**) rocket, (**C**) cabbage, (**D**) lettuce, (**E**) Chinese lettuce. The comparison between multiple samples was determined by One-way ANOVA using Tukey’ s test, and the different letters (a–d) indicate significant differences (*p* < 0.05). All the water content had no significant differences.

The water/ethanol extracts inhibited root length (RL) of vegetable seedlings in a dose-dependent relationship except rocket (Figure 3 and Figure 4 and Table 3). The SWE significantly promoted the shoot length (SL) of bok choy (1.25~10 mg/mL) and Chinese lettuce (2.5~10 mg/mL) and inhibited the other shoot length. The RL and SL of rocket first increased and then decreased with the change in the SEE concentration. The SEE significantly inhibited the SL of Chinese lettuce, cabbage, and lettuce. The SWE promoted the SL of bok choy and inhibited the RL, while the SEE had no significant effect on the SL and also inhibited the RL. Under treatment with SWE and SEE, the root-to-shoot ratio of bok choy decreased significantly. Stem thickness remained comparable to the control, and no obvious leaf yellowing occurred. These observations indicated that in response to the allelopathic extract, bok choy redistributed resources rather than mounting an escape response.

#### 2.1.3. Effects of Soil Extracts on Biomass Production

The dry weight (DW) and water content of seedlings were used to evaluate the allelopathic potential of soil extracts on five vegetable crops. The degree of inhibition varied among species (Figure 3 and Figure 4 and Table 4). The DW of Chinese lettuce and cabbage decreased in a dose-dependent manner with extracts. However, the DW of bok choy and lettuce unaffected by SWE. Interestingly, the DW of bok choy (5–10 mg/mL) and rocket (2.5–10 mg/mL) exhibited a significant increase by SEE. Regrettably, the seedlings were weighed as a whole in the experiment. In future experiments, the roots and above—ground parts will be weighed separately to calculate the biomass allocation ratio. The occurrence of short roots and long shoots will be analyzed in combination with the root—shoot ratio to explore its underlying mechanism.

#### 2.1.4. Effects of Extracts on RI Accumulated Value

**Figure 5:**
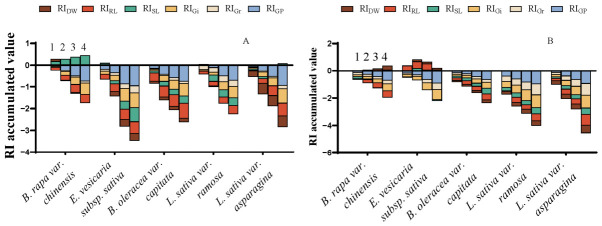
Effects of SWE and SEE on RI accumulated value. (**A**): SWE, (**B**): SEE. The adjacent columns from left to right (1, 2, 3, 4) in the same treatment represent concentrations of 1.25, 2.5, 5, and 10 mg/mL, respectively.

The comprehensive allelopathic effects of soil extracts were evaluated based on seed germination and seedling growth of five vegetable crops. Results indicated that both SWE and SEE inhibited five vegetable species, and the inhibition increased alongside dose-related dependent (Figure 5). Notably, the water extract exhibited the strongest inhibition on rocket, whereas the ethanol extract was most effective on lettuce. Conversely, both extracts demonstrated the weakest inhibitory effect on bok choy.

### 2.2. Effects of Soil Extracts on the Photosynthetic Pigments

**Figure 6:**
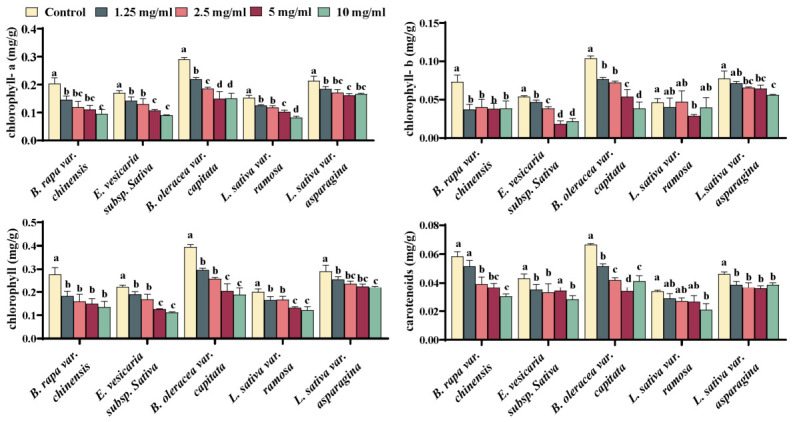
Effects of SWE on photosynthetic pigments. The comparison between samples was determined by One-way ANOVA using Tukey’ s test, and the different letters indicated significant differences (*p* < 0.05).

**Figure 7:**
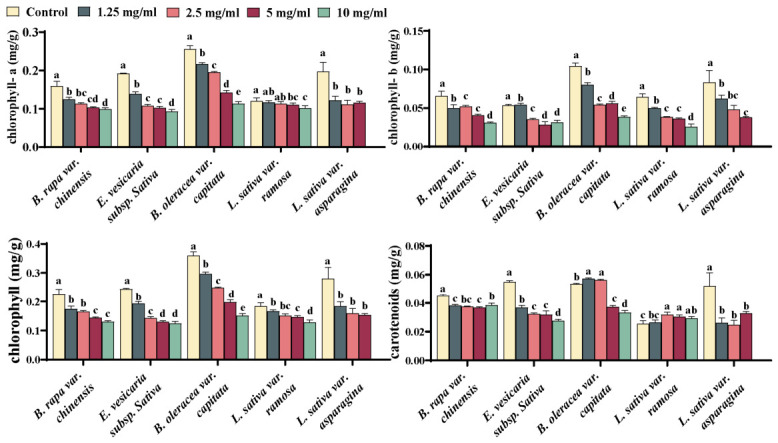
Effects of SEE on photosynthetic pigments. The comparison between samples was determined by One-way ANOVA using Tukey’ s test, and the different letters indicated significant differences (*p* < 0.05).

Photosynthetic pigment plays an important role in the growth and development of plants, and it is highly affected by allelochemicals [33]. Chlorophylls, plant pigments soluble in fat, consist of the following two primary constituents: chlorophyll-a (chl-a) and chlorophyll-b (chl-b), which act as photoreceptors in all photosynthetic organisms [34]. Both SWE and SEE reduced the content of total chlorophyll, as well as the contents of chlorophyll a, chlorophyll b, and carotenoids in five crops. The reduction in chlorophyll was positively correlated with the concentration of the extracts (Figure 6 and Figure 7).

### 2.3. Antioxidant Enzyme Activities

#### 2.3.1. MDA Content

**Figure 8:**
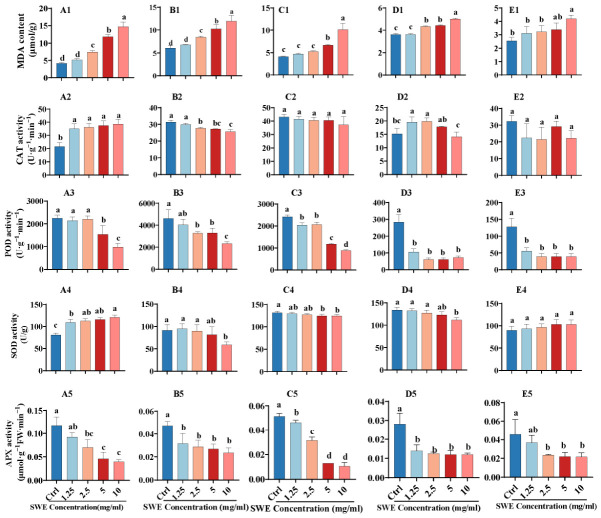
Physiological parameters of seedlings after treatment with SWE. (**A**) bok choy, (**B**) rocket, (**C**) cabbage, (**D**) lettuce, (**E**) Chinese lettuce. (**1**) MDA, (**2**) CAT, (**3**) POD, (**4**) SOD, (**5**) APX. The comparison between samples was determined by One-way ANOVA using Tukey’s test, and the different letters (a–d) indicated significant differences (*p* < 0.05).

**Figure 9:**
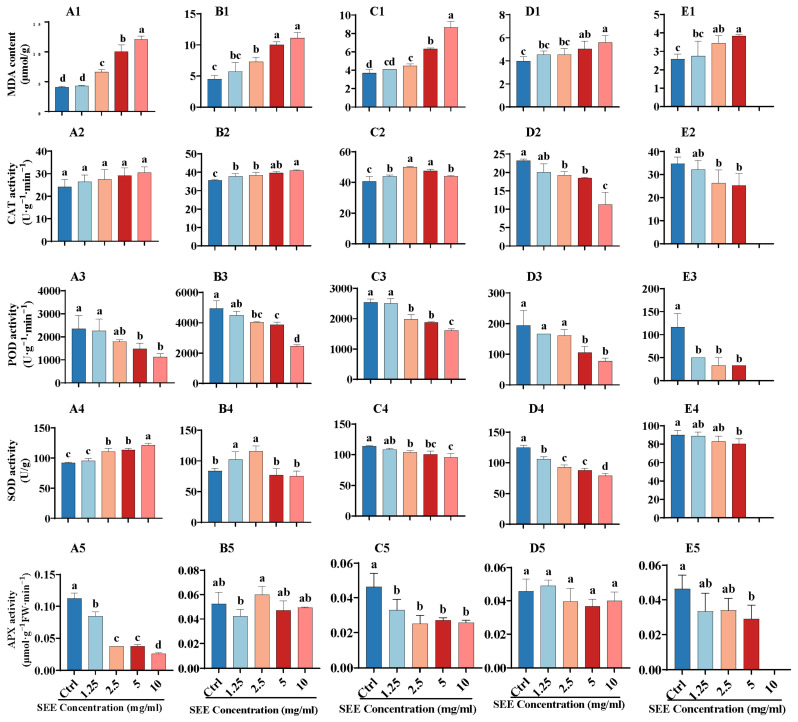
Physiological parameters of seedlings after treatment with SEE. (**A**) bok choy, (**B**) rocket, (**C**) cabbage, (**D**) lettuce, (**E**) Chinese lettuce. (**1**) MDA, (**2**) CAT, (**3**) POD, (**4**) SOD, (**5**) APX. The comparison between samples was determined by One-way ANOVA using Tukey’s test, and the different letters (a–d) indicated significant differences (*p* < 0.05).

Malondialdehyde, one of the end products of lipid peroxidation, is considered as an indicator of oxidative stress in cells [35]. It can represent the degree of cell membrane lipid peroxidation and plant stress injury, and the MDA content increases when plants are stressed [36]. Affected by the allelopathy of SWE and SEE, the content of MDA in all crops increased in a dose-dependent manner (Figure 8A and Figure 9A). Under SWE treatment, MDA increased in the order bok choy > cabbage > rocket > Chinese lettuce > lettuce, with respective rises of 256.7%, 146.4%, 96.3%, 65.4%, and 39.4%. Under SEE, the ranking was bok choy > rocket > cabbage > Chinese lettuce > lettuce, with increases of 199.6%, 146.5%, 133.57%, 48.6%, and 41.07%, respectively. These results show that both extracts induced oxidative stress, causing to MDA accumulation and damage to leaf cell membranes. Such membrane injury likely reduced photosynthetic efficiency and thereby impaired whole-plant growth.

#### 2.3.2. Antioxidant Enzyme Analysis

Plants have evolved an effective defense mechanism of antioxidant enzymes activities such as superoxide dismutase (SOD), peroxidase (POD), catalase (CAT), and ascorbic acid peroxidase (APX) to deal with oxidative damages [37,38]. The allelopathic effects of soil extracts significantly altered the enzyme activities of POD, CAT, APX, and SOD in five different crops (Figure 8 and Figure 9). Among the five crops, only bok choy displayed a consistent rise in SOD activity as the extract concentration increased (Figure 8D and Figure 9D). In comparison to the control group, each treatment group of SWE demonstrated a significant increase in SOD activity, with enhancements of 34.72%, 38.87%, 42.73%, and 49.26%, respectively. Similarly, the SOD activity of SEE exhibited a concentration-dependent increase, although it was lower than the treatment of SWE. SOD activity was significantly elevated in the three treatment groups by 18%, 25%, and 32%, respectively. In contrast, the lowest concentration of SEE did not achieve statistical significance. The SOD activity of rocket first increased and then decreased with increasing SEE. At 1.25 mg/mL and 2.5 mg/mL, the SOD activity increased significantly by 62.92% and 68.58% compared with the blank group (*p* < 0.05), while there was no significant difference at 5 mg/mL and 10 mg/mL. SOD of Chinese lettuce showed no significant change in the SWE, and the rest crops SOD decreased with increasing concentration of extracts.

The CAT activity in bok choy increased progressively with increasing extract concentrations (Figure 8B and Figure 9B). Compared with the control, CAT activity in the SWE groups increased significantly by 62.92%, 68.58%, 74.19%, 79.31%. In the SEE groups, CAT activity increased by 9.09%, 13.46%, 20.59%, and 25.99%, but these changes were not statistically significant. Overall, bok choy treated with SWE or SEE showed higher CAT and SOD activities than controls, indicating that it reduced oxidative damage through the elevation of SOD and CAT. The CAT activity in lettuce treated with SWE first increased and then decreased, with significant increases of 28.55% and 30.32% at 1.25 mg/mL and 2.5 mg/mL compared with the blank group. For rocket and cabbage treated with SEE, CAT activity increased significantly by 6.65%, 8.18%, 11.49%, 15.25%, 8.23%, 22.88%, 16.71%, and 7.99%. These results indicated that low-concentration SEE induced oxidative stress in rocket and cabbage, but the oxidative effect ceased once the extract concentration exceeded a threshold.

The POD activities of the five crops decreased with increasing concentration of water/ethanol extracts (Figure 8C and Figure 9C), and so did the APX activities, except for rocket and lettuce treated by SEE (Figure 8E and Figure 9E). The APX of Chinese lettuce and cabbage showed no significant change in the SEE.

## 3. Discussion

The results of this study showed that both SWE and SEE significantly inhibited germination of five vegetables, with SEE producing the stronger effect. This pattern suggests that the allelopathic activity arises from a complex mixture rather than a single constituent. Allelopathic activity is likely attributable to medium-polar triterpenoid saponins present in ginseng. The RI of germination rate under SEE treatment followed the following order: Chinese lettuce > rocket > lettuce > bok choy > cabbage, while under SWE treatment it was as follows: rocket > lettuce > Chinese lettuce > cabbage > bok choy. The results are similar to those of previous studies about the soil used for planting ginseng. It cannot be used again for ginseng or other crops due to allelopathy. The soil extracts had inhibitory effects on the seed germination of crops such as ginseng, cabbage, corn, mung bean, barley, and wheat [39,40,41,42,43,44,45,46,47,48,49]. The ethanol extracts significantly inhibited the hypocotyl growth of ginseng seeds, with a dose-dependent inhibition [41]. The water extract was separated using macroporous resin, and the methanol eluate exhibited allelopathic inhibitory effects on the germination rate, main root length, and fibrous root number of *Panax ginseng* and *Brassica campestris* seeds [42]. Both ginseng and soil extracts significantly inhibited the development of ginseng callus tissue [43].

This study found that soil extracts exhibited different allelopathy on the growth of five crop seedlings. Water and ethanol extract significantly inhibited both shoot and root growth in cabbage and lettuce, exhibiting a dose-dependent effect. In rocket, SEE initially promoted the root and shoot length then suppressed, whereas SWE produced consistent inhibition. These results indicate that the effects of the extracts are species specific. Allelopathy and its intensity depend on the recipient species and its sensitivity. A reasonable crop rotation system can be established based on the differences in species sensitivity.

SEE had no significant effect on the shoot length and dry weight of bok choy. This suggests that this species is relatively insensitive to SEE and may be suitable for subsequent crop rotation. Notably, SWE increased both shoot length and dry weight of bok choy, thereby reducing the root–shoot ratio. This phenomenon reflects a resource reallocation toward aboveground growth. Such a response aligns with an “escape” strategy rather than a “tolerance” strategy in plants under stress. While such an evasive approach may enhance short-term survival, its contribution to long-term stress resilience requires further investigation.

In the present experiment, each seedling was measured as a single unit; in future experiments, roots and shoots will be sampled separately. To elucidate the mechanism of the reduced root-to-shoot ratio, we will measure hormonal concentrations and the activities of oxidative enzymes.

The results showed that both SEE and SWE reduced the contents of chlorophyll and carotenoids in five crops. Previous work had confirmed that the allelochemical coumarin affected photosynthesis by reducing the chlorophyll content in the leaves of *Lolium perenne* [44]. In addition, benzoic acid and cinnamic acid inhibited root length and also reduced chlorophyll content in soybean [45]. The decrease may be related to the degradation of chlorophyll structure caused by accumulation of ROS in the extracts, which is similar to the maize in drought–stressed conditions [46]. The inhibition or degradation of photosynthetic pigments is one of the key mechanisms through which allelopathic substances suppress the growth of recipient plants.

The study revealed a consistent and notable increase in MDA content in the leaves of five receptor crops with escalating extract concentrations. This finding indicates that the extracts induce oxidative stress and compromise the integrity of cell membranes. Concurrently, the levels of chlorophyll and carotenoids decreased. It suggests that membrane lipid peroxidation may indirectly diminish the content of photosynthetic pigments by damaging chloroplast membranes.

Abiotic stresses induce reactive oxygen species (ROS) that cause oxidative stress in plants. A high level of ROS generated during abiotic stresses can cause injury to plant cells. To protect themselves, the plants alleviate oxidative stress by regulating the antioxidant system. Major ROS include singlet oxygen, superoxide radical, hydrogen peroxide, and hydroxyl radical in plants [47]. ROS has dual functionality in plants. Mild levels of ROS are considered as signals and achieve stress tolerance. And at higher concentrations, ROS can cause the denaturation of plasma membrane proteins, resulting in electrolyte leakage [48]. ROS can lead to cellular damage and programmed cell death [49,50]. Photosynthesis is also severely affected by oxidative stress [51].

The enzymatic components for detoxification of ROS include a lot of enzymes such as SOD, CAT, APX, and glutathione peroxidase (GPX) [52,53]. The SOD enzyme is considered to be one of the major enzymes for scavenging the stress-induced free-radical O_2_•^−^ in plants. It catalyzes the dismutation of O_2_•^−^ into H_2_O_2_ [54]. The product H_2_O_2_ is further detoxified by APX, GPX, and CAT [47]. The CAT enzyme is present in all cellular compartments in plants, such as mitochondria, the cytosol, and chloroplasts. It has a high Km value for H_2_O_2_ compared with APX; therefore, CAT is more active at high H_2_O_2_ concentrations than APX [55]. They catalyze H_2_O_2_ into water and oxygen in the cells exposed to environmental stress, and its catalytic activity does not require reducing agents [56]. APX is a member of the plant type heme peroxidase superfamily. The expression levels of APX are modulated in response to various types of abiotic stress [57,58].

Allelopathic stress disrupts the homeostasis of reactive oxygen species (ROS) in plants and activates their antioxidant defenses. The concentration-dependent increase in the activities of SOD and CAT suggests that bok choy has developed a defensive response to stress. Conversely, the decline in POD and APX suggests a limited capacity to mitigate ROS accumulation. Furthermore, the concurrent rise in MDA content signifies that oxidative damage surpasses the crop’s ability to repair itself. This finding is partially similar to the results of a study in *Nicotiana plumbaginifolia* [59], which reported that chemical treatment could enhance the activities of CAT and SOD in sunflower seedlings. A previous study showed that the accumulation of POD increased in the roots but decreased in the leaves of tea plants under high concentrations of Al stress [60]. POD and APX activities decreased under extracts and related to the concentration in bok choy, consistent with the decline in POD of tea leaves. As stress from SEE intensified, rocket exhibited a dynamic antioxidant response. Initially, SOD activity increased, followed by a subsequent decline, while CAT activity and MDA levels consistently rose. In contrast, APX activity remained relatively stable, and POD activity decreased. These findings indicate that the antioxidant defense in rocket is inadequate to counteract the oxidative damage induced by SEE. In summary, the extract disrupts the balanced regulation of antioxidant enzymes in crops, causing inadequate ROS clearance and membrane lipid peroxidation. This may be one of the important physiological mechanisms by which allelochemicals inhibit the growth of recipient plants.

This study lacks chromatographic identification of allelochemicals in ginseng soil extracts, limiting mechanistic interpretation to physiological responses. Based on prior reports, phenolic acids, ginsenosides, and fatty acids are candidate compounds. Future HPLC/LC-MS/GC-MS analysis is needed to establish direct chemical-biological links.

It is worth noting that the continuous cropping obstacles of ginseng are multifactorial. This study focuses on the hydroponic method to determine the effects of extracts on seed germination and seedling growth, eliminating interference from microorganisms and pathogens. But other factors such as soil-borne pathogens and imbalanced microbial communities also play a key role. Therefore, future crop rotation research should combine pathogen abundance, microbial community structure, and allelopathy. At present, the screening in the laboratory is a preliminary screening of candidate crops in rotation; Field validation under natural conditions is necessary to capture the full complexity of continuous cropping obstacles.

## 4. Materials and Methods

### 4.1. Soil Material

The soil was collected from Fusong County, Jilin Province (127°01′–128°06′ E, 41°42′–42°49′ N), following five years of ginseng cultivation. It was then sieved through a 40-mesh screen and air-dried at room temperature for one week.

### 4.2. Water Extract and Ethanol Extract Preparation

The dried soil was extracted with 95% ethanol by heating reflux method three times, and then filtered. Ethanol was recovered under reduced pressure to obtain SEE. Similarly, the dried soil was extracted with water by boiling three times, and then evaporated to obtain SWE. The crude extracts were diluted to different concentrations of 1.25, 2.5, 5, and 10 (mg/mL) solutions.

### 4.3. Plant Seeds

Seeds of the five vegetable crops were purchased from Jilin Lida Seed Company (Table 5).

### 4.4. Determination of Seed Germination

The experiment evaluated the effects of SWE and SEE on the seed germination of five vegetable crops using the flat dish method. The seeds were disinfected with 0.2% sodium hypochlorite solution for 10 min and then rinsed three times with sterile water. Fifty seeds were equidistantly placed in petri dish (90 mm diameter) with two layers filter paper. Then, 5 mL water/ethanol extracts at concentrations of 1.25, 2.5, 5, and 10 mg/mL were added to the petri dish. Sterile water was used as the blank control and added 1 mL every 24 h, keeping the filter paper moist. All treatments were performed with three biological replicates. The dishes were cultured in constant temperature incubator (Hangzhou Allsheng Instruments Co., Ltd., HangZhou, China) at 25 ± 0.5 °C. Seeds were considered germinated when the radicle was at least 2 mm long. After 7 days, GR, GP, GI, RI, and SE were determined and calculated.

### 4.5. Experiment of Seedling Growth

Germinated seeds were placed in culture cups, and water/ethanol extracts of 1.25 mg/mL, 2.5 mg/mL, 5 mg/mL, and 10 mg/mL were added to each cup. Sterile water served as the blank control, with three replicated for each group. The culture cups were placed in a constant-temperature incubator at 25 °C ± 0.5 °C. After 21 days, SL, RL, and biomass were determined according to the literature [25].

### 4.6. Determination of Photosynthetic Pigments Content

As a primary photosynthetic pigment, chlorophyll combats oxidative stress by inhibiting the production of ROS and scavenging ROS. Carotenoids are one of natural pigments in plants, which can neutralize reactive oxygen radicals and reduce oxidative stress damage in cells. Extraction and quantification of chlorophylls a, b, total chlorophyll, and carotenoids were performed according to the method of Lichtenthaler and Wellburn [61]. The chlorophylls and total carotenoid content were expressed as mg/g, respectively.

### 4.7. Antioxidant Enzymatic Activities Experiment

Antioxidant enzyme analysis was conducted on the leaves of seedlings. The activity of SOD was determined by the ability to inhibit the photochemical reduction in NBT [62,63]. The activity of POD was measured by the guaiacol colorimetric method [64]. The activities of CAT and ascorbic acid peroxidase (APX) were determined by ultraviolet spectrophotometry [65,66,67]. The content of malondialdehyde was determined by the TBA method [68,69].

### 4.8. Statistical Analysis

Germination rate (GR) = (number of germinated seeds/total number of seeds) × 100% [70]; Germination percentage (GP) = (the number of germinated seeds at 3nd day/total number of seeds) × 100) [71]; Germination Index (GI) = ∑Gt/Dt, Gt is the number of all germinated seeds at a given day, and Dt is the corresponding day number [72]; Response index (RI) = 1 − C/T (T ≥ C), RI = C/T − 1 (T < C), RI > 0 indicates allelopathic stimulation, and RI< 0 indicates allelopathic inhibition [73]. Synthetic allelopathy (SE) = RI accumulate value = RIGR + RIGP + RIGI + RIDW + RIRL + RISL [74]. Data were presented as means ± standard error. Analysis of variance (ANOVA) and multiple comparisons were carried out with the GraphPad Prism (v11) and SPSS (v24.0). Statistically significant differences were set at *p* values equal to or lower than 0.05.

## 5. Conclusions

Both SWE and SEE demonstrated concentration-dependent allelopathic inhibition of the seed germination and seedling growth in five crops, with SEE showing stronger effects. Species-specific sensitivity was observed; rocket was most affected by SWE, lettuce by SEE, and bok choy showed the greatest tolerance to both. Interestingly, SWE promoted shoot height while suppressing root length in bok choy and Chinese lettuce, and SEE induced a hormetic effect on rocket. The increased dry biomass in bok choy under ethanolic treatment, despite reduced root-to-shoot radio, suggests a shift in biomass allocation from roots to shoots as an escape strategy. These findings highlight the importance of extract type, concentration, and recipient species in evaluating allelopathic potential.

The concentration-dependent reduction in photosynthetic pigments in five crops under SWE and SEE stress confirms that damage to photosynthesis is a mechanism of allelopathic inhibition. The significant increase in MDA levels indicates that lipid peroxidation occurs during crop growth, resulting in membrane damage. Additionally, species-specific variations in antioxidant enzyme activity were observed. In bok choy, a sustained upregulation of SOD and CAT underlies the physiological basis for tolerance. At the same time, the low sensitivity of rocket and cabbage under SEE stress is attributed to the activation of CAT.

We conclude that the allelopathic effects of the extracts on the five crops operate through a multi-target strategy. This strategy includes the direct reduction in photosynthetic pigments and the disruption of the reactive oxygen species (ROS) scavenging network. The tolerance observed in bok choy is associated with the effectiveness of SOD/CAT pathway. In contrast, the susceptibility of the other crops arises from irreversible oxidative damage due to an imbalance in antioxidant defenses.

## Figures and Tables

**Table 1:** The Effect of Water Extracts on the GP, GR, and GI of Five Crops.

Crops	EC (mg/mL)	SWE GP (%)	SWE GR (%)	SWE GI
***Brassica rapa* var. *chinensis* (L.) Kitam.**	Control	60.88 ± 5.74 ^a^	92.44 ± 3.01 ^a^	34.73 ± 3.47 ^a^
	1.25	57.55 ± 2.77 ^a^	91.11 ± 2.69 ^a^	33.50 ± 1.80 ^a^
	2.5	45.33 ± 0.66 ^b^	87.56 ± 1.01 ^ab^	28.54 ± 2.32 ^b^
	5	29.11 ± 4.72 ^c^	88.00 ± 2.31 ^ab^	23.10 ± 2.83 ^c^
	10	15.33 ± 1.15 ^d^	83.11 ± 5.55 ^b^	16.91 ± 2.96 ^d^
***Eruca vesicaria* subsp. *sativa* (M.) Thellung**	Control	46.22 ± 2.77 ^a^	89.78 ± 0.77 ^a^	27.64 ± 2.05 ^a^
	1.25	36.89 ± 0.38 ^b^	80.67 ± 0.67 ^b^	23.84 ± 1.79 ^b^
	2.5	29.11 ± 1.38 ^c^	78.89 ± 3.08 ^c^	20.69 ± 1.23 ^c^
	5	5.56 ± 2.03 ^d^	71.11 ± 2.69 ^d^	11.67 ± 1.28 ^d^
	10	1.33 ± 0.67 ^e^	61.11 ± 5.05 ^e^	8.75 ± 0.64 ^e^
***Lactuca sativa* var. *asparagina* L.H.Bailey ex Holub**	Control	70.00 ± 5.03 ^a^	90.00 ± 0.67 ^a^	37.76 ± 2.34 ^a^
	1.25	64.44 ± 4.01 ^a^	86.22 ± 2.34 ^b^	35.47 ± 3.06 ^a^
	2.5	51.11 ± 3.15 ^b^	84.44 ± 0.38 ^b^	29.56 ± 3.30 ^b^
	5	32.44 ± 6.74 ^c^	83.56 ± 1.92 ^b^	24.20 ± 3.01 ^c^
	10	3.78 ± 1.01 ^d^	76.00 ± 1.15 ^c^	13.19 ± 1.43 ^d^
***Brassica oleracea* var. *capitata* L.**	Control	54.07 ± 5.01 ^a^	92.96 ± 5.25 ^a^	19.29 ± 1.46 ^a^
	1.25	45.93 ± 1.28 ^b^	89.26 ± 2.57 ^ab^	17.46 ± 2.55 ^a^
	2.5	30 ± 5.09 ^c^	88.52 ± 4.63 ^ab^	14.01 ± 2.68 ^b^
	5	21.85 ± 3.57 ^d^	82.22 ± 1.11 ^b^	11.41 ± 1.02 ^c^
	10	12.96 ± 1.7 ^e^	82.59 ± 3.39 ^b^	8.55 ± 2.05 ^d^
***Lactuca sativa* var. *ramosa* Hort.**	Control	37.78 ± 4.44 ^a^	88.00 ± 2.9 ^a^	24.06 ± 2.38 ^a^
	1.25	37.33 ± 3.52 ^a^	70.20 ± 3.36 ^b^	22.69 ± 2.21 ^ab^
	2.5	33.11 ± 3.01 ^a^	68.00 ± 3.06 ^bc^	20.93 ± 1.91 ^b^
	5	18.44 ± 4.34 ^b^	64.20 ± 2.78 ^bc^	15.16 ± 2.76 ^c^
	10	10.89 ± 2.34 ^c^	62.90 ± 4.07 ^c^	11.61 ± 2.69 ^d^

EC: Extract Concentration, GP: Germination Potential, GR: Germination Rate, GI: Germination Index, a–e indicate significant differences (*p* < 0.05).

**Table 2:** The Effect of Ethanol Extracts on the GP, GR, and GI of Five Crops.

Crops	EC (mg/mL)	SEE GP (%)	SEE GR (%)	SEE GI
***Brassica rapa* var. *chinensis* (L.) Kitam.**	Control	56.00 ± 1.76 ^a^	90.00 ± 1.15 ^a^	32.14 ± 3.09 ^a^
	1.25	47.56 ± 5.05 ^ab^	74.67 ± 4.16 ^b^	27.64 ± 3.18 ^b^
	2.5	39.78 ± 4.91 ^b^	76.44 ± 10.36 ^ab^	25.64 ± 2.76 ^bc^
	5	30.44 ± 7.43 ^c^	75.33 ± 8.51 ^b^	22.75 ± 4.40 ^c^
	10	15.56 ± 1.39 ^d^	65.56 ± 6.19 ^b^	15.79 ± 2.40 ^d^
***Eruca vesicaria* subsp. *sativa* (M.) Thellung**	Control	45.56 ± 3.67 ^a^	87.78 ± 1.68 ^a^	28.21 ± 2.81 ^a^
	1.25	37.11 ± 7.31 ^ab^	79.11 ± 2.78 ^b^	23.24 ± 3.53 ^b^
	2.5	31.11 ± 2.04 ^b^	77.33 ± 1.33 ^b^	21.02 ± 1.74 ^b^
	5	15.56 ± 9.19 ^c^	66.67 ± 7.57 ^c^	13.83 ± 5.16 ^c^
	10	3.33 ± 1.15 ^d^	47.33 ± 4.16 ^d^	6.95 ± 2.36 ^d^
***Lactuca sativa* var. *asparagina* L.H.Bailey ex Holub**	Control	64.67 ± 1.76 ^a^	87.78 ± 2.52 ^a^	34.81 ± 1.49 ^a^
	1.25	54.44 ± 5.82 ^a^	74.22 ± 3.91 ^b^	29.13 ± 2.84 ^b^
	2.5	38.44 ± 8.88 ^b^	60.00 ± 4.06 ^c^	21.71 ± 3.70 ^c^
	5	20.89 ± 5.39 ^c^	44.89 ± 4.91 ^d^	13.64 ± 4.01 ^d^
	10	2.67 ± 2.91 ^d^	13.33 ± 1.33 ^e^	2.52 ± 1.56 ^e^
***Brassica oleracea* var. *capitata* L.**	Control	53.70 ± 5.13 ^a^	88.52 ± 2.80 ^a^	19.21 ± 2.14 ^a^
	1.25	44.81 ± 0.64 ^ab^	78.15 ± 4.49 ^ab^	16.78 ± 1.66 ^ab^
	2.5	40.00 ± 6.67 ^bc^	78.51 ± 7.14 ^ab^	15.72 ± 2.82 ^bc^
	5	29.63 ± 4.49 ^cd^	72.59 ± 7.14 ^b^	13.19 ± 2.28 ^cd^
	10	18.52 ± 8.98 ^d^	70.37 ± 5.25 ^b^	10.74 ± 2.05 ^d^
***Lactuca sativa* var. *ramosa* Hort.**	Control	36.44 ± 0.38 ^a^	85.56 ± 1.02 ^a^	22.83 ± 1.73 ^a^
	1.25	21.33 ± 7.69 ^b^	52.00 ± 7.33 ^b^	12.85 ± 3.44 ^b^
	2.5	13.33 ± 0.67 ^c^	47.78 ± 4.44 ^b^	9.66 ± 1.95 ^c^
	5	5.33 ± 2.31 ^d^	36.67 ± 2.91 ^c^	4.90 ± 1.40 ^d^
	10	0.67 ± 0.00 ^d^	17.33 ± 6.77 ^d^	1.60 ± 0.80 ^e^

EC: Extract Concentration, GP: Germination Potential, GR: Germination Rate, GI: Germination In-dex, a–e indicate significant differences (*p* < 0.05).

**Table 3:** The Effect of Extracts on SL and RL of Five Seedings.

Crops	EC (mg/mL)	SWE Shoot Length	SWE Root Length	SEE Shoot Length	SEE Root Length
***Brassica rapa* var. *chinensis* (L.) Kitam.**	Control	1.95 ± 0.10 ^d^	7.18 ± 0.30 ^a^	2.66 ± 0.16 ^a^	7.50 ± 0.31 ^a^
	1.25	2.41 ± 0.16 ^c^	6.23 ± 0.39 ^b^	2.25 ± 0.11 ^a^	7.13 ± 0.29 ^a^
	2.5	2.74 ± 0.02 ^c^	5.57 ± 0.2 ^c^	2.55 ± 0.21 ^a^	5.87 ± 0.36 ^b^
	5	3.15 ± 0.22 ^b^	4.63 ± 0.02 ^d^	2.63 ± 0.46 ^a^	4.84 ± 0.87 ^c^
	10	3.53 ± 0.28 ^a^	4.43 ± 0.19 ^d^	2.92 ± 0.50 ^a^	4.01 ± 0.31 ^c^
***Eruca vesicaria* subsp. *sativa* (M.) Thellung**	Control	2.39 ± 0.13 ^a^	2.50 ± 0.12 ^a^	2.37 ± 0.23 ^bc^	2.37 ± 0.13 ^b^
	1.25	2.65 ± 0.06 ^b^	1.92 ± 0.21 ^b^	2.27 ± 0.12 ^bc^	3.87 ± 0.61 ^a^
	2.5	2.04 ± 0.06 ^c^	1.60 ± 0.14 ^c^	2.95 ± 0.27 ^a^	4.70 ± 0.53 ^a^
	5	1.51 ± 0.14 ^d^	1.30 ± 0.04 ^d^	2.62 ± 0.19 ^ab^	4.20 ± 0.53 ^a^
	10	0.87 ± 0.12 ^e^	1.14 ± 0.10 ^d^	2.21 ± 0.08 ^d^	2.57 ± 0.14 ^b^
***Lactuca sativa* var. *asparagina* L.H.Bailey ex Holub**	Control	2.92 ± 0.20 ^bc^	2.90 ± 0.13 ^a^	3.24 ± 0.08 ^a^	2.74 ± 0.09 ^a^
	1.25	2.75 ± 0.10 ^bc^	2.77 ± 0.13 ^a^	2.82 ± 0.10 ^b^	2.31 ± 0.22 ^ab^
	2.5	2.67 ± 0.11 ^c^	2.27 ± 0.67 ^b^	2.38 ± 0.19 ^c^	1.73 ± 0.17 ^b^
	5	3.04 ± 0.21 ^ab^	1.60 ± 0.07 ^c^	2.22 ± 0.16 ^c^	1.73 ± 0.58 ^b^
	10	3.21 ± 0.06 ^a^	1.19 ± 0.05 ^d^	1.71 ± 0.28 ^d^	0.57 ± 0.25 ^c^
***Brassica oleracea* var. *capitata* L.**	Control	3.38 ± 0.09 ^a^	3.24 ± 0.20 ^a^	3.41 ± 0.21 ^a^	3.70 ± 0.13 ^a^
	1.25	3.05 ± 0.19 ^b^	2.05 ± 0.23 ^b^	3.00 ± 0.15 ^b^	2.99 ± 0.53 ^b^
	2.5	2.68 ± 0.14 ^c^	1.63 ± 0.09 ^c^	2.70 ± 0.06 ^c^	2.73 ± 0.10 ^b^
	5	2.52 ± 0.11 ^c^	1.51 ± 0.17 ^c^	2.67 ± 0.16 ^c^	2.51 ± 0.08 ^bc^
	10	2.24 ± 0.09 ^d^	1.04 ± 0.15 ^d^	2.05 ± 0.09 ^d^	2.08 ± 0.35 ^c^
***Lactuca sativa* var. *ramosa* Hort.**	Control	3.30 ± 0.07 ^a^	2.40 ± 0.12 ^a^	2.99 ± 0.27 ^a^	2.01 ± 0.14 ^a^
	1.25	3.20 ± 0.10 ^a^	2.10 ± 0.11 ^b^	2.26 ± 0.11 ^b^	1.92 ± 0.31 ^a^
	2.5	2.35 ± 0.07 ^b^	1.90 ± 0.05 ^c^	2.07 ± 0.09 ^b^	1.25 ± 0.10 ^b^
	5	2.20 ± 0.05 ^c^	1.70 ± 0.07 ^d^	2.10 ± 0.09 ^b^	1.35 ± 0.23 ^b^
	10	2.15 ± 0.11 ^c^	1.50 ± 0.09 ^e^	1.60 ± 0.10 ^c^	1.00 ± 0.04 ^b^

EC: Extract Concentration, a–e indicate significant differences (*p* < 0.05).

**Table 4:** The Effect of Extracts on Fresh Weight and Dry Weight of Five Seedings.

Plant Species	EC (mg/mL)	SWE FW (mg)	SWE DW (mg)	SEE FW (mg)	SEE DW (mg)
***Brassica rapa* var. *chinensis* (L.) Kitam.**	Control	29.30 ± 8.13 ^b^	2.75 ± 0.85 ^a^	38.77 ± 1.11 ^a^	3.23 ± 0.15 ^c^
	1.25	37.10 ± 2.57 ^ab^	3.07 ± 0.23 ^a^	36.40 ± 1.64 ^a^	3.28 ± 0.20 ^c^
	2.5	38.70 ± 4.38 ^ab^	2.69 ± 0.70 ^a^	36.70 ± 3.20 ^a^	3.61 ± 0.21 ^bc^
	5	41.25 ± 1.71 ^a^	2.61 ± 0.62 ^a^	37.80 ± 2.96 ^a^	3.92 ± 0.29 ^b^
	10	45.65 ± 8.09 ^a^	2.74 ± 0.00 ^a^	40.53 ± 2.89 ^a^	4.61 ± 0.27 ^a^
***Eruca vesicaria* subsp. *sativa* (M.) Thellung**	Control	32.85 ± 2.47 ^ab^	3.30 ± 0.35 ^a^	36.93 ± 1.03 ^b^	3.47 ± 0.28 ^b^
	1.25	35.90 ± 4.20 ^a^	3.32 ± 0.36 ^a^	35.73 ± 1.99 ^b^	3.51 ± 0.26 ^b^
	2.5	27.88 ± 2.52 ^bc^	2.66 ± 0.31 ^ab^	41.10 ± 1.50 ^a^	4.03 ± 0.11 ^a^
	5	26.86 ± 0.31 ^bc^	2.52 ± 0.54 ^ab^	38.60 ± 1.70 ^ab^	4.02 ± 0.10 ^a^
	10	22.88 ± 6.17 ^c^	2.23 ± 0.51 ^b^	36.70 ± 3.00 ^b^	4.09 ± 0.12 ^a^
***Lactuca sativa* var. *asparagina* L.H.Bailey ex Holub**	Control	24.83 ± 1.66 ^a^	2.49 ± 0.12 ^a^	26.33 ± 1.65 ^a^	2.66 ± 0.11 ^a^
	1.25	20.57 ± 3.42 ^ab^	1.85 ± 0.12 ^b^	21.03 ± 2.53 ^b^	1.97 ± 0.05 ^b^
	2.5	15.50 ± 2.21 ^c^	1.30 ± 0.13 ^c^	17.76 ± 2.45 ^b^	1.81 ± 0.14 ^b^
	5	16.83 ± 2.40 ^bc^	1.35 ± 0.08 ^c^	16.80 ± 2.23 ^b^	1.73 ± 0.10 ^b^
	10	16.90 ± 1.93 ^bc^	1.26 ± 0.10 ^c^	10.27 ± 2.68 ^c^	1.20 ± 0.16 ^c^
***Brassica oleracea* var. *capitata* L.**	Control	42.43 ± 2.40 ^a^	4.55 ± 0.08 ^a^	40.83 ± 2.51 ^a^	4.04 ± 0.16 ^a^
	1.25	40.87 ± 2.11 ^ab^	4.09 ± 0.14 ^b^	36.90 ± 1.11 ^ab^	3.66 ± 0.10 ^b^
	2.5	38.20 ± 1.18 ^bc^	3.96 ± 0.07 ^b^	33.70 ± 1.49 ^bc^	3.54 ± 0.21 ^b^
	5	37.73 ± 1.10 ^bc^	3.88 ± 0.04 ^c^	31.93 ± 1.69 ^cd^	3.46 ± 0.10 ^bc^
	10	36.47 ± 2.03 ^c^	3.79 ± 0.08 ^c^	28.80 ± 2.71 ^d^	3.22 ± 0.59 ^c^
***Lactuca sativa* var. *ramosa* Hort.**	Control	13.50 ± 1.30 ^a^	1.17 ± 0.01 ^a^	18.40 ± 1.37 ^a^	1.82 ± 0.15 ^a^
	1.25	12.90 ± 0.61 ^ab^	1.23 ± 0.06 ^a^	15.10 ± 1.41 ^b^	1.48 ± 0.12 ^b^
	2.5	10.80 ± 0.40 ^bc^	1.14 ± 0.70 ^a^	12.97 ± 1.64 ^bc^	1.35 ± 0.08 ^bc^
	5	10.70 ± 1.29 ^bc^	1.18 ± 0.15 ^a^	11.67 ± 1.46 ^c^	1.28 ± 0.15 ^bc^
	10	10.00 ± 0.76 ^c^	1.20 ± 0.09 ^a^	10.47 ± 0.83 ^c^	1.19 ± 0.06 ^c^

EC: Extract Concentration, FW: Fresh Weight, DW: Dry Weight, a–d indicate significant differences (*p* < 0.05).

**Table 5:** Origin and Cultivar of seeds.

English Name	Latin Scientific Name	Cultivar
Bok Choy	*Brassica rapa* var. *chinensis* (L.) Kitam.	Creamy and Fast-Growing Chinese Broccoli
Rocket	*Eruca vesicaria* subsp. *sativa* (M.) Thellung	Big Leaf Rocket
Chinese lettuce	*Lactuca sativa* var. *asparagina* L.H.Bailey ex Holub	Xiao Hua Ye
Cabbage	*Brassica oleracea* var. *capitata* L.	Zhonggan No.11
Lettuce	*Lactuca sativa* var. *ramosa* Hort.	Glass Lettuce

## Data Availability

The datasets generated during and analyzed during the current study and all plant materials are available from the corresponding author on reasonable request.

## References

[1] Wang C.N., Zhao D.Q., Wang L.S., Wang J. (2021). Study on the two-way immune regulation mechanism and clinical application of Ginseng and Compound Preparation. Lishizhen Med. Mater. Medica Res..

[2] Li Q.Y., Tang J.X., Liu S.W., Xu P., Zhou J.Q., Yin F., Mi J.W., Yu L., Wang L.N., Bi Y.F. (2022). Protective effects of black ginseng fermentation on fatigue stress-induced oxidative damage and comparison with white ginseng, red ginseng and ginseng berry. Food Sci..

[3] Liu F.X., Lin Z.X., Zhang H.L., Zhang Z.Q., Yang K.Q. (2019). Analysis of anti-fatigue mechanism and potential targets of ginseng. China J. Chin. Mater. Medica.

[4] Kim J.H. (2018). Pharmacological and medical applications of *Panax ginseng* and ginsenosides: A review for use in cardiovascular diseases. J. Ginseng Res..

[5] Luo L.M., Shi Y.N., Jiang Y.N., Zhan J.H., Qin L., Chen N.H. (2017). Advance in components with antitumor effect of *Panax ginseng* and their mechanisms. Chin. Tradit. Herb. Drugs.

[6] Liu L., Xu F.R., Wang Y.Z. (2020). Traditional uses, chemical diversity and biological activities of *Panax* L. (Araliaceae): A review. J. Ethnopharmacol..

[7] Committee C.P. (2020). Pharmacopoeia of the People’s Republic of China.

[8] Bian X., Xiao S., Zhao Y., Zhang L. (2020). Comparative analysis of rhizosphere soil physiochemical characteristics and microbial communities between rusty and healthy ginseng root. Sci. Rep..

[9] Xiao C., Yang L., Zhang L., Liu C., Han M. (2016). Effects of Cultivation Ages and Modes on Microbial Diversity in the Rhizosphere Soil of *Panax Ginseng*. J. Ginseng Res..

[10] Li C.W., Chen G.Z., Zhang J.L., Bai X.F., Zhu P., Hou Y.P., Zhang X.X. (2020). Long-Term Changes in Soil Properties and Microbial Communities are Continuous Cropping Obstacles Associated with American Ginseng (*Panax Quinquefolius* L.) Cultivation. Preprints.

[11] Behdarvandi B., Goodwin P.H. (2023). Effect of Soil and Root Extracts on the Innate Immune Response of American Ginseng (*Panax Quinquefolius*) to Root Rot Caused by Ilyonectria Mors-Panacis. Plants.

[12] Yang M., Zhang X.D., Xu Y.G., Mei X.Y., Jiang B.B., Liao J.J. (2015). Autotoxic Ginsenosides in the Rhizosphere Contribute to the Replant Failure of *Panax notoginseng*. PLoS ONE.

[13] Liu C., Xia R., Tang M., Chen X., Zhong B., Liu X., Bian R., Yang L., Zheng J., Cheng K. (2022). Improved ginseng production under continuous cropping through soil health reinforcement and rhizosphere microbial manipulation with biochar: A field study of *Panax ginseng* from Northeast China. Hortic. Res..

[14] Li J., Chen Y., Zhao G., Chen Y., Zhang N., Yu D. (2024). Herbal materials used as soil amendments alleviate root rot of *Panax ginseng*. Sci. Rep..

[15] Peng N., Bi Y.M., Jiao X.L., Zhang X.M., Li J.F., Wang Y., Yang S.S., Liu Z.Q., Gao W.W. (2024). A soil fumigant increases American ginseng (*Panax quinquefolius* L.) survival and growth under continuous cropping by affecting soil microbiome assembly: A 4-year in situ field experiment. Microbiol. Spectr..

[16] Jin J.R., Ma Q.L., Wang X.Q., Dou S., Wang Q.B. (2010). Study on Effect of Ecology Method on *Panax Ginseng* Continuous Cropping Ground Soil Improvement. J. Gensing Rearch.

[17] Inderjit (2005). Soil microorganisms: An important determinant of allelopathic activity. Plant Soil.

[18] Margaret M. (2007). Rhizosphere allelopathy. Allelopath. J..

[19] Zhou T.T., Zhan Y., Li Q., Wang E.P., Huang X., Chen C.B. (2025). Research progress on allelochemicals types and mechanisms of medicinal plants in the genus Panax. Spec. Wild Econ. Anim. Plant Res..

[20] Bi X.B., Yang J.X., Gao W.W. (2010). Autotoxicity of phenolic compounds from the soil of American ginseng (*Panax quinquefolium* L.). Allelopath. J..

[21] Jiao X.L., Bi X.B., Gao W.W. (2015). Allelopathic effect of p-coumaric acid on American ginseng and its physiological mechanism. Acta Ecol. Sin..

[22] Li Z.B., Zhou R.J., Fu J.F. (2018). Allelopathic effects of phenolic acids from Ginseng rhizosphere soil on *Cylindrocarpon destructans* (Zinss) Scholten. Allelopath. J..

[23] He C.N., Gao W.W., Yang J.X., Bi W., Zhang X.S., Zhao Y.J. (2009). Identification of autotoxic compounds from fibrous roots of *Panax quinquefolium* L.. Plant Soil.

[24] Jiang J.L., Yu M., Li L., Li Y.K., Cao X.L. (2019). Relationship between root rot and accumulation of ginsenosides in *Panax quinquefolius*. Chin. Tradit. Pat. Med..

[25] Li Q., Zhang L.X., Guan T.Z., Xu Y.H., Chen C.B. (2020). Allelopathic effects of ginsenoside Rg1 on seed germination and seedling growth of *Panax ginseng*. Allelopath. J..

[26] Zhang Q.J., Geng Y.Q., Gao Y.G., Zhang L.X. (2012). Effects of ginsenosides on seedling growth and development of *Panax ginseng*. Chin. Tradit. Herb. Drugs.

[27] Jia F.G., Chang F., Guan M., Jia Q.G., Sun Y., Li Z. (2023). Effects of rotation and Bacillus on the changes of continuous cropping soil fungal communities in American ginseng. World J. Microbiol. Biotechnol..

[28] Wang Y., Zhang H., Zhang Y., Fei J., Rong X., Peng J., Luo G. (2023). Crop Rotation-Driven Changes in Rhizosphere Metabolite Profiles Regulate Soil Microbial Diversity and Functional Capacity. Agric. Ecosyst. Environ..

[29] Bian X., Yang X., Zhang K., Zhai Y., Li Q., Zhang L., Sun X. (2023). Potential of Medicago Sativa and Perilla Frutescens for Overcoming the Soil Sickness Caused by Ginseng Cultivation. Front. Microbiol..

[30] Zhang J., Zhou D., Yuan X., Xu Y., Chen C., Zhao L. (2022). Soil Microbiome and Metabolome Analysis Reveals Beneficial Effects of Ginseng–Celandine Rotation on the Rhizosphere Soil of Ginseng-Used Fields. Rhizosphere.

[31] Yu H.Q., Yu H.R., Liu S.Y., Zhang X., Lei T., Wang Y.Q., Cheng L., Han M., Yang L.M. (2023). Effects of ginseng platycodon grandiflorum rotation on soilmicroecology in ginseng field. J. Jilin Agric. Univ..

[32] Fan D., Zhao Z., Wang Y., Ma J., Wang X. (2022). Crop-type-driven changes in polyphenols regulate soil nutrient availability and soil microbiota. Front. Microbiol..

[33] Ghimire B., Hwang M., Sacks E., Yu C., Kim S., Chung I. (2020). Screening of Allelochemicals in *Miscanthus sacchariflorus* Extracts and Assessment of Their Effects on Germination and Seedling Growth of Common Weeds. Plants.

[34] Gebregziabher B., Zhang S., Ghosh S., Shaibu A., Azam M., Abdelghany A., Qi J., Agyenim-Boateng K.G., Htway H., Feng Y. (2022). Origin, Maturity Group and Seed Coat Color Influence Carotenoid and Chlorophyll Concentrations in Soybean Seeds. Plants.

[35] Duric M., Subotic A., Prokic L., Trifunovic-Momcilov M., Cingel A., Vujicic M., Milosevic S. (2020). Morpho-Physiological and Molecular Evaluation of Drought and Recovery in *Impatiens walleriana* Grown Ex Vitro. Plants.

[36] Zhang Y., Cao G., Li X., Piao Z. (2023). Effects of Exogenous Ergothioneine on *Brassica rapa* Clubroot Development Revealed by Transcriptomic Analysis. Int. J. Mol. Sci..

[37] Ahmad S., Wang G.Y., Muhammad I., Chi Y.X., Zeeshan M., Nasar J., Zhou X.B. (2022). Interactive Effects of Melatonin and Nitrogen Improve Drought Tolerance of Maize Seedlings by Regulating Growth and Physiochemical Attributes. Antioxidants.

[38] Ulhassan Z., Huang Q., Gill R.A., Ali S., Mwamba T., Ali B., Hina F., Zhou W. (2019). Protective mechanisms of melatonin against selenium toxicity in *Brassica napus*: Insights into physiological traits, thiol biosynthesis and antioxidant machinery. BMC Plant Biol..

[39] Li Y., Zhu D.L., Huang X.F., Ding W.L. (2008). Inhibition of different soil extracts on growth of ginseng seeds. Chin. Tradit. Herb. Drugs.

[40] Meng X.R., Huang X., Li Q., Wang E., Chen C.B. (2023). Application of UPLC-Orbitrap-HRMS targeted metabolomics in screening of allelochemicals and model plants of ginseng. J. Plant Physiol..

[41] Li Y., Huang X.F., Ding W.L., Zhang R. (2008). Allelopathic effects of soil extracts on the growth of ginseng seeds and its chemical composition. Ecol. Environ..

[42] Chen C.B., Liu J.Y., Wang Y.Y., Yan S., Xu S.Q. (2006). Allelopathy of ginseng rhizosphere and its effect on germination of seed. J. Jilin Agric. Univ..

[43] Chen C.B., Xu S.Q., Liu J.Y., Li C.Y., Wang Y.P., Zhang L.X. (2006). Study on the influence of ginseng allelopathy on growth of ginseng callus. J. Tongji Univ. Med. Sci..

[44] Yao D.D., Wang J.Y., Zhou Q., Tang Q., Zhao G.Q., Wu C.X. (2017). Effect of coumarin on *Italian ryegrass* seed germination and seedling growth. Acta Prataculturae Sin..

[45] Baziramakenga R., Simard R.R., Leroux G.D. (1994). Effects of benzoic and cinnamic acids on growth, mineral composition, and chlorophyll content of soybean. J. Chem. Ecol..

[46] Ibrahim A.E., Abd El M.T., Abohamid Y., Abdallah H., El-Saadony M., AbuQamar S., El-Tarabily K., Abdou N., Abdou N. (2022). Exogenously Applied Proline Enhances Morph-Physiological Responses and Yield of Drought-Stressed Maize Plants Grown Under Different Irrigation Systems. Front. Plant Sci..

[47] Wang P., Liu W.C., Han C., Wang S., Bai M.Y., Song C.P. (2023). Reactive oxygen species: Multidimensional regulators of plant adaptation to abiotic stress and development. J. Integr. Plant Biol..

[48] Savchenko G.E., Klyuchareva E.A., Abramchik L.M., Serdyuchenko E.V. (2002). Effect of Periodic Heat Shock on the Inner Membrane System of Etioplasts. Russ. J. Plant Physiol..

[49] Lushchak V. (2015). Free radicals, reactive oxygen species, oxidative stresses and their classifications. Ukr. Biochem. J..

[50] Foyer C.H. (2018). Reactive oxygen species, oxidative signaling and the regulation of photosynthesis. Environ. Exp. Bot..

[51] Wang G.P., Zhang X.Y., Li F., Luo Y., Wang W. (2010). Overaccumulation of glycine betaine enhances tolerance to drought and heat stress in wheat leaves in the protection of photosynthesis. Photosynthetica.

[52] Murshed R., Lopez-Lauri F., Sallanon H. (2008). Microplate quantification of enzymes of the plant ascorbate–glutathione cycle. Anal. Biochem..

[53] Gill S.S., Tuteja N. (2010). Reactive oxygen species and antioxidant machinery in abiotic stress tolerance in crop plants. Plant Physiol. Biochem..

[54] Dumanović J., Nepovimova E., Natić M., Kuča K., Jaćević V. (2021). The significance of reactive oxygen species and antioxidant defense system in plants: A concise overview. Front. Plant Sci..

[55] Palma J.M., Mateos R.M., López-Jaramillo J., Rodríguez-Ruiz M., González-Gordo S., Lechuga-Sancho A.M., Corpas F.J. (2020). Plant catalases as NO and H_2_S targets. Redox Biol..

[56] Sharma P., Jha A.B., Dubey R.S., Pessarakli M. (2012). Reactive oxygen species, oxidative damage, and antioxidative defense mechanism in plants under stressful conditions. J. Bot..

[57] Anjum N.A., Gill S.S., Gill R., Hasanuzzaman M., Duarte A.C., Pereira E., Ahmad I., Tuteja R., Tuteja N. (2014). Metal/metalloid stress tolerance in plants: Role of ascorbate, its redox couple, and associated enzymes. Protoplasma.

[58] Ozyigit I.I., Filiz E., Vatansever R., Kurtoglu K.Y., Koc I., Öztürk M.X., Anjum N.A. (2016). Identification and Comparative Analysis of H_2_O_2_-Scavenging Enzymes (Ascorbate Peroxidase and Glutathione Peroxidase) in Selected Plants Employing Bioinformatics Approaches. Front. Plant Sci..

[59] Singh A., Singh D., Singh N.B. (2015). Allelopathic activity of *Nicotiana plumbaginifolia* at various phenological stages on sunflower. Allelopath. J..

[60] Xu Q.S., Wang Y., Ding Z.T., Fan K., Ma D.X., Zhang Y.L., Yin Q. (2017). Aluminum induced physiological and proteomic responses in tea (*Camellia sinensis*) roots and leaves. Plant Physiol. Biochem..

[61] Lichtenthaler H.K., Wellburn A.R. (1983). Determinations of total carotenoids and chlorophylls a and b of leaf extracts in different solvents. Analysis.

[62] Giannopolitis C.N., Ries S.K. (1972). Superoxide dismutase I. Occurrence in higher plants. Plant Physiol..

[63] Cakmak I., Marschner H. (1992). Magnesium Deficiency and High Light Intensity Enhance Activities of Superoxide Dismutase, Ascorbate Peroxidase, and Glutathione Reductase in Bean Leaves 1. Plant Physiol..

[64] Zhu H.F., Li X.F., Zhai W., Liu Y., Gao Q.Q., Liu J.Q., Ren L., Chen H.Y., Zhu Y.Y. (2017). Effects of low light on photosynthetic properties, antioxidant enzyme activity, and anthocyanin accumulation in purple pak-choi (*Brassica campestris* ssp. Chinensis Makino). PLoS ONE.

[65] Jiang M.Y., Zhang J.H. (2001). Effect of Abscisic Acid on Active Oxygen Species, Antioxidative Defence System and Oxidative Damage in Leaves of Maize Seedlings. Plant Cell Physiol..

[66] Aebi H. (1984). Catalase in vitro. Methods in Enzymology: Oxygen Radicals in Biological Systems.

[67] Zhang X.Q., Liang Y.J., Kai Z., Wu C.X., Yang L.T., Li Y.R. (2016). Influence of inoculation of *Leifsonia xyli* subsp. Xyli on photosynthetic parameters and activities of defense enzymes in sugarcane. Sugar Tech.

[68] Bates L.S., Waldren R.P., Teare I.D. (1973). Rapid determination of free proline for water-stress studies. Plant Soil.

[69] Kapoor D., Tiwari A., Sehgal A., Landi M., Brestic M., Sharma A. (2019). Exploiting the Allelopathic Potential of Aqueous Leaf Extracts of *Artemisia absinthium* and *Psidium guajava* against *Parthenium hysterophorus*, a Widespread Weed in India. Plant.

[70] Wang C.Y., Xiao H.G., Zhao L.L., Liu J., Wang L., Zhang F., Shi Y.C., Du D.L. (2016). The allelopathic effects of invasive plant *Solidago canadensis* on seed germination and growth of Lactuca sativa enhanced by different types of acid deposition. Ecotoxicology.

[71] Wang C.Y., Jiang K., Zhou J.W., Liu J. (2017). Allelopathic suppression by Conyza canadensis depends on the interaction between latitude and the degree of the plant’s invasion. Acta Bot. Bras..

[72] Zhao T.L., Deng X.L., Xiao Q.Z., Han Y.F., Zhu S.J., Chen J.H. (2020). IAA priming improves the germination and seedling growth in cotton (*Gossypium hirsutum* L.) via regulating the endogenous phytohormones and enhancing the sucrose metabolism. Ind. Crops Prod..

[73] Williamson G.B., Richardson D. (1988). Bioassays for allelopathy: Measuring treatment responses with independent controls. J. Chem. Ecol..

[74] Zhang Y., Liu S.L., Xiao D., Chen Z.G., Ma Z.Y., Mu Y.G. (2024). The inhibitory potential of green manure return on the germination and seedling growth of *Eleusine indica* L.. Front. Plant Sci..

